# Adults with Perinatally Acquired HIV; Emerging Clinical Outcomes and Data Gaps

**DOI:** 10.3390/tropicalmed9040074

**Published:** 2024-04-03

**Authors:** Merle Henderson, Sarah Fidler, Caroline Foster

**Affiliations:** 1 900 Clinic, Imperial College Healthcare NHS Trust, London W2 1NY, UK; m.henderson@imperial.ac.uk (M.H.); s.fidler@imperial.ac.uk (S.F.); 2 Department of Infectious Diseases, Imperial College London, Imperial College NIHR BRC, London W2 1NY, UK; 3 Department of Paediatric Infectious Diseases, Imperial College Healthcare NHS Trust, London W2 1NY, UK

**Keywords:** perinatally acquired HIV, young adults, mortality, morbidity, antiretroviral therapy, drug resistance, mental health, sexual health

## Abstract

In resourced settings, adults living with perinatally acquired HIV are approaching the 5th decade of life. Their clinical and psychological outcomes highlight potential future issues for the much larger number of adolescents growing up with HIV in sub–Saharan Africa, and will inform the development of appropriate healthcare services. Lifelong exposure to HIV, and increasingly to antiretroviral therapy throughout growth and development, contrasts with adults acquiring HIV in later life. This review describes the clinical outcomes for adults living with perinatally acquired HIV including post transition mortality, morbidity and retention in care. Rates of viral suppression, drug resistance and immunological function are explored. Co-morbidities focus on metabolic, cardiovascular, respiratory and bone health with quality-of-life data including neurocognitive functioning and mental health. Sexual and reproductive health including vaccine-preventable disease and the prevention of onward transmission to partners and infants are considered. The data gaps and future research questions to optimise outcomes for this emerging adult cohort are highlighted.

## 1. Introduction and Epidemiology

Since 1990, global data estimate that more than 11 million children have been born with perinatally acquired HIV, with 85% in sub-Saharan Africa [1]. Survivors of the perinatal epidemic born before 2006 are now adults, aged 18 years and older. Since the peak in 2000, annual rates of global new infections in infants has fallen from more than 530,000 (CI—360,000–830,000) to 130,000 (CI—90,000–210,000) in 2022 [1]. Whilst rates of childhood infection have reduced by 58% since 2010, with an estimated 3.4 million new infections prevented, gains appear to have plateaued in recent years [2,3]. In 2022, 82% (64–98%) of pregnant or breast feeding people living with HIV had access to antiretroviral therapy (ART), meaning 220,000 pregnant women could not access ART for themselves or to prevent transmission to their infants [2,3]. Without treatment, 50% of infants will die before their second birthday, although up to 10% will survive into adolescence, with minimal symptoms, reflecting the diverse disease progression of paediatric HIV [4]. AIDS-related deaths peaked in children in 2004 at 360,000 and whilst deaths fell by 64% from 2010 to 84,000 in 2022, this represented 13% of global AIDS-related deaths, yet children make up just 4% of people living with HIV [3]. When compared to adults, children are both less likely to receive ART (57% [44–78%] versus 77% [65–89%]) and to attain viral suppression (46% versus 71%). Of the estimated 1.5 million children under 15 years of age living with HIV, 660,000 still do not have access to treatment [3]. 

Yet despite the global inequalities in both the prevention of perinatal transmission and access to suppressive ART in childhood, increasing numbers of adolescents living with perinatally acquired HIV (LWPaHIV) are surviving into adult life. They transition into adult services where they join a cohort of young people who acquired HIV non-perinatally (nPaHIV) during adolescence/early adulthood. An estimated 480,000 (255,000–760,000) of the total 1.3 million new infections in 2022 occurred in young people aged 10-24 years [3]. In high income settings (HIC) with access to universal screening in pregnancy and ART, rates of perinatal transmission have fallen to below 0.5% for the past decade, with the majority of transmissions now occurring in women who seroconvert during pregnancy or breast feeding [5,6]. In such settings with reassuringly small numbers of newly diagnosed infants, the numbers of children accessing paediatric care continue to fall, with the majority of people LWPaHIV having transitioned to adult services [7]. In regions such as Europe and the United States, where people LWPaHIV have been accessing ART since the late 1990s, the oldest survivors are now approaching their 5th decade of life [8]. Whilst the survivor bias may mean that those born in the pre-ART era, now in their 30s and representing a unique cohort with slow progression, favourable host genetics and immune responses, many of those that are now in their 20s started ART in infancy [4]. The issues faced by this latter population, which are now embedded in adult care, are more likely to be reflective of the emerging adolescent cohorts globally and are extremely important in identifying the consequences of lifelong HIV and ART exposure throughout postnatal, childhood and adolescent development. 

This review describes the published outcomes for adults aged ≥18 years living with perinatally acquired HIV, and it identifies data gaps and areas for future research.

## 2. Cascade of Care 

In 2020, the Joint United Nations Programme for HIV/AIDS (UNAIDS) established the 95-95-95 targets for 2030, whereby 95% of all people living with HIV would be aware of their HIV status, 95% of those diagnosed to be living with HIV would be accessing ART and 95% of those accessing ART would achieve viral suppression [3]. By the end of 2022, UNAIDS estimates for all ages suggested that targets achieved had reached 86-89-93, and whilst there was a marked improvement from 71-67-83 in 2015, the outcomes for children aged 0-14, the majority of whom are infected perinatally, remained starkly poorer, as they were estimated at 63-91-81 [3]. For those over 15 years, disaggregating global data by the route of infection, perinatal versus other, becomes complex and hampered by inadequate data linkage, as adolescents move from paediatric to adult care with much of the outcome data for adults (age ≥ 18 years) disaggregated by the route of transmission (PaHIV versus nPaHIV) limited to single country cohorts [9].

The period of transition from paediatric to adult care has been associated with higher rates of loss to follow up (LTFU), with around 75% people LWPaHIV retained in care for 4 years post transition [10]. A tertiary centre in Thailand reported post transition outcomes for 101 youth, with 97% perinatally infected and a median age at transition of 20 years [10]. At 2 years post transition, 87% were retained in care, with 77% being virally suppressed (HIV viral load (VL) < 50 c/mL). Factors associated with viral suppression were a later age at transition (≥20 years: aOR 4.38, 95% CI 1.41–13.65) and being on first line therapy (aOR 6.05, 95% CI 1.55–23.58) [10], reflecting better outcomes amongst those who were already well engaged in care and on treatment. A total of 30% had one or more co-morbidities, most frequently diabetes/dyslipidaemia (15%) and cognitive impairment (8%). Amongst more than 4200 Kenyan 10–24 year olds, almost half being people LWPaHIV, the mortality was highest in the 10–14 year olds on ART for less than 2 years with advanced WHO staging (mortality incidence per 100 person-years: 3.31 (95% CI: 1.66–6.62)), which probably reflected a late diagnosis of perinatal HIV [11]. Conversely, LTFU was highest in the 20–24 year olds (asHR: 2.52 (95% CI: 1.91–3.33); *p* < 0.001) and was greatest (>40%) in those newly enrolled in care. Across the cohort, LTFU was lower in perinatal HIV (sHR: 0.35 (95% CI: 0.25–0.49). Young adults LWPaHIV in HIC do not necessarily fare better, although data are variable. In a US youth cohort, one in five were LTFU in the 12 months after their 22nd birthday, with no difference in the route of transmission, PaHIV versus nPaHIV; rates of LTFU increased with age and retention was poorer in adult services when compared to adolescent clinics [12,13]. Effective interventions to enhance the retention in care for young adults living with HIV remain critical to prevent both individual mortality and onward transmission to partners and offspring [14].

European PaHIV cohorts, despite reporting lower rates of LFTU, report an all-cause mortality in adult care of 2.3–4.9/1000, which is at least ten times their aged and country-matched uninfected peers [7,8,15,16,17]. In a stark contrast to adults acquiring HIV in later life, causes of death in PaHIV in all settings were almost all due to end-stage AIDS events, primarily opportunistic infections, with HIV-associated malignancies and a small number of suicides reported [7,8,15,16,17,18]. In a multicentre US cohort of people LWPaHIV, mortality increased with age and was highest in adults aged 18–30 years (*n* = 967), with those aged 20–29 having a mortality rate that was 12 times the national age-matched average [19]. The health utilisation, including hospital admission and AIDS events, was also higher in US adults LWPaHIV when compared to both younger children and aged-matched nPaHIV adults [20]. The mortality in the International Epidemiologic Databases to Evaluate AIDS (LeDEA) cohort of people LWPaHIV in Africa, Asia Pacific, South America and the Caribbean was highest in infancy, then decreased with age during later childhood, only to rise again in early adulthood [21]. Whilst not disaggregated by the route of infection (PaHIV versus nPaHIV) and without outcome data, rates of malignancy, the majority being AIDS-related, in a South African national surveillance study, were worryingly high, with an incidence of 61/100,000 people per year; associated with lower CD4 counts and included cervical carcinoma in 15–19 year olds [22]. Mortality post transition to adult care for adults LWPaHIV is predictable; the associated lower CD4 count exiting paediatric care and a prior AIDS diagnosis in childhood highlighted the continuing impact of early childhood events much later in adult life [7,18,21]. A summary of data gaps and recommendations for improving the cascade of care is highlighted in Table 1.

## 3. Viral Suppression and Acquired Drug Resistance

Rates of viral suppression are lower in adolescents (10–19 years) and young adults (20–24 years) than in adults over 25 years, with a global meta-analysis showing adherence rates of 62% in those age 10–24 years; however, the outcome varies widely by setting [23]. In a US adult perinatal cohort (*n* = 444; ≥18 years old; median age: 21.3; 64% female; 70% of a black ethnicity), 44% were on suppressive ART, with poorer adherence associated with perceived low levels of social support in those who had yet to transition to adult services [24]. A European cohort study from the Netherlands (*n* = 1331) confirmed that younger adults aged 18-24 years were more likely to experience virological failure than those aged 25–30 years (OR 2.34 [95%CI 1.48–3.71] PaHIV and 1.27 [95%CI 1.07–1.50] nPaHIV) [25]. Viral suppression was achieved by 81% PaHIV aged 18–24 years versus 93% nPaHIV, the latter approaching rates of suppression in Dutch adults > 25 years of 95%. Rates of suppression in young adults LWPaHIV in the UK were remarkably similar to the Dutch cohort, with a median age of 22.9 years, 85% black African ethnicity, and 81% of whom were suppressed [8]. In a Spanish multicentre cohort (*n* = 332), at a transition median age of 18 years, 73.3% were supressed; this improved to 85.6% after three years in adult care [15]. The improvement was only observed in earlier calendar years (1997–2006) and may in part reflect changes in ART over time; it also reflects that the transition to adult care does not have to be associated with adverse outcomes if it is well managed [15]. 

Rates of triple class resistance are higher in young adults LWPaHIV when compared to nPaHIV; this is a reflection of many more years of ART exposure. In an Italian cohort, people LWPaHIV aged 18 to 30+ years, rates of dual, triple and four-class resistance, were 41%, 20% and 6%, respectively, with 7% having at least one mutation conferring integrase resistance by 2019 [26]. Comparable rates of triple class resistance were observed in Spanish adults LWPaHIV (15%), although lower rates were observed in a comparable UK cohort (6%) [8,27]. Triple class resistance was reported in pregnant women LWPaHIV, with the potential for a transmitted drug resistance in infected infants making treatment extremely challenging [28]. Whilst most adults LWPaHIV still have effective oral ART regimens, many continue to struggle with adherence to daily medication. The extensive prior exposure to non-nucleoside reverse transcriptase inhibitors (NNRTI), the mainstay of paediatric ART in many settings prior to the roll out of dolutegravir, has resulted in the extensive NNRTI resistance that renders the currently approved long-acting injectable options unsuitable [29]. The desire for, and potential benefits of, ultra-long-acting ART delivery systems cannot be underestimated for young people, some of whom have struggled with daily oral medication for their entire lives [30]. A summary of data gaps and recommendations for improving viral suppression is highlighted in Table 2.

## 4. Neurocognitive, Mental Health and Quality of Life outcomes in Adults Living with PaHIV

HIV-associated neurological disorders cause a wide range of manifestations that vary across the life course, from infantile HIV encephalopathy to HIV-associated dementia in adults [31]. Central nervous system opportunistic infections such as tuberculous and cryptococcal meningitis, cytomegalovirus, toxoplasmosis and progressive multifocal leukoencephalopathy add to the burden. Prior to the roll out of ART, half of children born with HIV experienced neurological manifestations, from hypertonic diplegia and the severe neurocognitive impairment observed with infantile HIV encephalopathy, through to delayed language in preschool children, as well as attention deficits and behavioural disorders in school-aged children [31]. Early suppressive ART reduces but does not fully ameliorate the risk of neurological sequelae; however, the importance of early infant diagnosis and treatment in protecting the developing brain cannot be underestimated [32,33,34,35]. Studies throughout childhood and adolescence, when well matched with HIV-exposed uninfected (HEU) controls to adjust for environmental, socioeconomic and psychological stressors, show that ever having had a CDC C diagnosis, particularly HIV encephalopathy, is associated with poorer neurocognitive functioning [36,37]. The neurocognitive outcomes for adults LWPaHIV comprise two groups; those with a more slowly progressing disease surviving in the pre-ART era and those presenting with rapidly progressive disease from the late 1990s in HIC with access to early ART, albeit with older and more toxic regimens. Data from adult cohorts LWPaHIV in the US and Europe suggest that differences in neurocognitive structure and functioning, including learning and memory, which are important for education and employment, persist longitudinally and are most apparent in those with prior CDC C diagnoses, often occurring 2 decades previously [38,39,40,41]. Early findings suggesting accelerated aging in young adults LWPaHIV highlight the importance of longitudinal follow up of very long-term brain health in adulthood [40,42]. 

The impact of LWPaHIV on quality of life (QOL) in adulthood is multifactorial, and differs from youth who acquire HIV in adolescence due to the impact of living in a family where multiple generations live with HIV. With suppressive ART, health-related QOL has improved markedly; however, high rates of double orphanhood continue to impact adult life. For young adults LWPaHIV in India (mean age: 18.9), almost two thirds were double orphans, having further impacts on education, with 58% dropping out of school and from employment [43]. However, in the US, markers of resilience, including high school graduation, tertiary education and current employment, were comparable between young adults aged 19–27 LWPaHIV and HEU youth [44]. Others, mainly single centre cohorts, report lower rates of educational attainment and employment in both groups (PaHIV and HEU) when compared to age-matched population norms [31,38,45]. In Botswana (PaHIV, ages 18–30), the overall health-related QOL was good, with outcomes significantly associated with education, employment, viral suppression and self-reported illness [46]. However, those with disability, neurocognitive impairment, care leavers, young parents and the unemployed were the most vulnerable, reporting a poorer QOL [47].

Mental health disorders are associated with a reduction in quality of life, with their onset peaking in adolescence and young adulthood, including for those living with HIV [48]. The majority of studies suggest that rates of anxiety and depression (18–40%) are comparable to HEU youth, although above that of the general age-matched population, suggesting that social, familial and system level stressors may play more of a role than HIV itself [49,50,51,52,53,54]. HIV stigma impacts mental health, QOL, engagement in healthcare and adherence to ART. For a longitudinal cohort of people LWPaHIV in the US, with a mean age of 24.9 (SD 2.56) at last follow up, an older age predicted worse mental health outcomes and was associated with perceived HIV stigma [55]. To quote the authors, “it is sobering that as we approach the third decade of the epidemic, HIV stigma remains a persistent reality that compromises the mental health of people with PaHIV–underscoring the renewed importance of carefully integrated mental health and stigma-reduction programming and addressing barriers to accessing these services.” HIV-related stigma causes disclosure-related anxiety and prevents youth sharing their status with family, friends and sexual partners for fear of rejection [56,57]. Status sharing can be particularly complex for those LWPaHIV, with the potential for an inadvertent disclosure of parental/sibling status, although, encouragingly, intervention studies to support disclosure for this population are emerging [58]. 

Suicide is a leading cause of death in young adults globally, with prior suicide attempts being predictors of subsequent suicide. In a US longitudinal study, 22% of youth LWPaHIV, with an average age of 24.6 years, reported one or more suicide attempts in more than 10 years of follow up; associated with depression and behavioural disorders [59]. Rates of attempted suicide were twice that of HEU youth, although, reassuringly, no deaths by suicide were reported in either group [59]. Subsequent follow up from the same cohort identified HIV-stigma, past-year arrest and pregnancy as additional risk factors for suicidality, whilst hope for the future and religiosity were protective [60,61]. In a cohort of young adults LWPaHIV in Thailand, with a median age of 21.7 years, viral non-suppression was associated with a lifetime suicide attempt and reported in 7.9% [62]. Suicide was given as the cause of death in 2 of 11 young people with PaHIV in the UK, both aged 22 years, one with prior suicidal ideation and the second with a history of psychosis [17].

As for adults LWnPaHIV, early data suggested that rates of psychosis may be higher when compared to age-matched rates in the general population [63]. Risk factors for psychosis in the general population include family history, substance use, poverty, black ethnicity, learning disability, childhood social care placement, migration and any prior central nervous system infection/injury, with multiple risk factors common in adults LWPaHIV. Consistent associations are observed between mental health difficulties, substance use and poorer adherence to ART, with higher rates of LFTU [64,65]. Substance use by youth living with HIV has been associated with increased risk behaviours, including unprotected sex [66,67]. Having a recent substance-use disorder was associated with an increased risk of being a victim of intimate partner violence (IPV) in a US cohort for both PaHIV and HEU young adults, citing an exceedingly high life time prevalence of 85% and 65% within the past year [38]. In a South African cohort of young women LWPaHIV, 41% of those aged 20–24 reported life time IPV, 32% within the last year associated with increasing childhood adversity [68]. IPV, within the past year, was associated with high-risk sex, pregnancy, depression and substance use. More widely, experiencing repeated episodes of violence and/or psychological abuse including HIV stigma was associated with higher rates of virological failure in Zambian youth [69]. The intersectionality of mental, physical and sexual health cannot be underestimated, particularly during the multiple developmental tasks of adolescence and early adult life. The global need for the integration of mental health care within HIV services for young adults LWHIV is paramount to enable the social and economic success of the next generation (Table 3).

## 5. Metabolic and Cardiovascular Health

### 5.1. Metabolic Health

Obesity is an increasing public health concern globally, with significant long-term health impacts, including cardiovascular disease, diabetes mellitus and musculoskeletal complications. Evidence suggests a high prevalence of obesity in people living with HIV [70], which may be related to ART-use and demographic characteristics as well as secondary to lifestyle factors [71]. Alterations in body fat composition have also been demonstrated in adults LWPaHIV, with a recent US study reporting higher rates of truncal fat deposition in those LWPaHIV (*n* = 70, median age of 26 and IQR—23–29), measured by waist to hip ratio and trunk to limb fat ratio, when compared to people without HIV (0.91 ± 0.1 vs. 0.84 ± 0.1 and 0.98 ± 0.3 vs. 0.76 ± 0.2, both *p* < 0.0001, respectively), who are associated with a chronic exposure to ART, particularly historic regimens that included the non-nucleoside reverse transcriptase inhibitors (NRTIs) stavudine (r = 0.36, *p* = 0.02) and didanosine (r = 0.39, 0.01) [72]. Increases in the waist to hip ratio were positively correlated with increases in triglycerides (r = 0.35, *p* = 0.03). While the prevalence of individuals who were either overweight (body mass index (BMI) > 25–29) or obese (BMI > 30) was similar to that of 47 HIV-negative controls (47% vs. 51% and 20% vs. 17%, respectively), those LWPaHIV had higher rates of metabolic syndrome (13% vs. 4%), which was defined as three of five abnormal measurements; elevated waist circumference, triglycerides, blood pressure, fasting glucose and/or elevated triglycerides or reduced HDL cholesterol [72]. Similarly, in a French study of 18–30 year olds LWPaHIV, 13.2% of men and 10.4% of women were identified with metabolic syndrome, compared to 10.6% and 1.7% in an HIV-negative control population, with increased central adiposity and abnormalities in lipid profiles demonstrated in those LWPaHIV [73]. These findings have been reflected in other low–middle and high income settings; in a study of 120 Thai young people LWPaHIV aged 15–25 years (mean age: 20.3 ± 2.6), 12 (10.6%) were identified with the metabolic syndrome [74], of which 92% were male, 50% were overweight and 67% had abnormal lipid profiles; a UK study of 85 young adults (15–24 years) LWPaHIV demonstrated evidence of the metabolic syndrome in 7% [75].

Weight gain secondary to integrase-strand transfer inhibitor (INSTI)-based ART regimens, particularly those containing dolutegravir (DTG) and the NRTI tenofovir alafenamide (TAF) have been reported in adults with HIV [76]. Data on weight gain in young adults LWPaHIV are conflicting and remain limited. A retrospective cohort analysis of weight gain in 38 children and youth LWPaHIV (aged 2–19 years; 95% Black ethnicity), initiating INSTIs in the US, demonstrated an increased rate of change in the BMI for the age z-score over a median follow up period of 527 days (IQR—477–625) [77]. In contrast, a 52-week study of 64 South African adolescents LWPaHIV on suppressive ART (median age at baseline: 13.5 years (IQR—12.5–14.4)) demonstrated no significant change in the BMI z-score in a subset of 30 individuals that had switched to a DTG-containing ART regimen [78]. Both cholesterol and triglycerides were lower in the DTG-ART group, and the prevalence of hepatic steatosis, measured by transient elastography, decreased from 17% to 3%; however, this may have been secondary to the cessation of lopinavir/ritonavir and/or zidovudine-containing ART, and starting tenofovir disoproxil, in the majority of participants [78]. These findings were reflected in a US study of 66 adults LWPaHIV (mean age: 26.67 ± 4.79 and 92% Caucasian ethnicity), 45 of which switched to an INSTI-based ART regimen, with no significant difference in weight gain between groups over a median follow up of 9 years (range: 2–10) [79].

Non-alcoholic fatty liver disease (NAFLD), an accumulation of triglycerides within hepatocytes, is a leading cause of chronic liver disease globally, and linked to traditional risk factors such as obesity and dyslipidaemia. A high burden of NAFLD exists among people living with HIV, with a recent systematic review and meta-analysis of 43 studies demonstrating a 33.9% pooled prevalence in this population [80]. Data from a US study of adults LWPaHIV (*n* = 46; mean age: 27 ± 3.1) suggest higher rates of NAFLD when compared to demographically matched HIV-negative controls (*n* = 20; 33% vs. 10%, respectively) [81]. Individuals LWPaHIV also had higher levels of metabolic abnormalities when compared to those without HIV, including dyslipidaemia, body fat deposition, insulin resistance and inflammatory biomarkers (CRP; d-dimer) [81]. 

### 5.2. Hypertension

Hypertension is defined as a blood pressure ≥ 140/90 mmHg on repeated examination [82]. People living with HIV may be at a higher risk of hypertension, when compared to people without HIV, due to traditional risk factors for cardiovascular disease (CVD), as well as HIV-related factors such as vascular inflammation, lipodystrophy and in relation to ART usage [83]. Prevalence estimates of hypertension in those LWPaHIV vary between 3 and 31% [84,85,86,87], with a limited number of studies (*n* = 3) including young adults over 18 years of age. In a cross-sectional retrospective study of mostly African American young people LWPaHIV (*n* = 108) aged 18–29 years, matched by age and sex to young people LWnPaHIV (*n* = 108) and HIV-uninfected young adults (*n* = 108), the prevalence of hypertension was 23% in PaHIV vs. 10% in nPaHIV and 8% in HIV-uninfected young adults [85]. In this study, hypertension was defined as a clinical documentation of a systemic hypertension diagnosis or prescription of an antihypertensive medication directed towards blood pressure management [85]. These findings were reflected in a US single centre retrospective study of 18–25 years olds (*n* = 159), in which the prevalence of hypertension was 16.3%, albeit based on a single blood pressure reading [88]. Poorly controlled hypertension is a risk factor for CVD, chronic kidney disease and stroke [84]; early diagnosis and intervention may be critical to reducing the future risk of CVD. 

### 5.3. Cardiovascular Disease

Young people LWPaHIV may also be at a higher risk of CVD due to structural vascular changes [87,89]. Increased carotid intima media thickness, a biomarker of subclinical atherosclerosis, has been demonstrated in children and young people LWPaHIV (aged 2.5–24 years), compared to age-matched HIV-negative controls [87]. These findings were independent of HIV viral load suppression and may indicate a higher risk of CVD [87]. In a pooled analysis of >2000 people living with HIV and HIV-negative controls, after controlling for demographic and cardiovascular risk-related variables, carotid intima media thickness was found to be greatest in children and young adults living with HIV (6–29 years; *n* = 221) when compared to HIV-negative age-matched controls (*n* = 58), with sensitivity analyses strengthened by perinatal HIV status [90]. A study of coronary vascular disease using a CT coronary angiography and coronary vessel wall MRI in asymptomatic young people LWPaHIV aged 15–29 years vs. HIV negative controls evidenced an increased right coronary arterial wall thickening, which was associated with increased duration of ART, hyperlipidemia and smoking [91]. In a Thai study of 120 young people LWPaHIV (15 to <25 years) 36% had a raised high sensitivity CRP, an acute-phase protein associated with systemic inflammation and used as a predictor of cardiovascular risk [74]. Chronic exposure to certain ART agents may also lead to cardiovascular complications. The cumulative effects of protease inhibitors can lead to abnormalities in lipid profiles, which may predispose to cardiovascular disease [92]. Data from adults living with HIV have highlighted an association between the recent use of abacavir and an increased risk of myocardial infarction [93], which may be related to platelet activation and endothelial dysfunction [94,95]. No data exist on prior/current abacavir use and CVD risk in adults LWPaHIV. Long-term survivors of HIV-associated malignancies who received cardiotoxic chemotherapy may require additional monitoring for cardiovascular complications; anthracycline-induced and impaired systolic function, once diagnosed, is often irreversible [96]. A summary of data gaps and recommendations for improving metabolic and cardiovascular health are highlighted in Table 4.

## 6. Respiratory Health

With access to widespread, effective ART, the incidence of AIDS-related respiratory infections has declined. Despite this, chronic lung disease (CLD) remains prevalent in children and young people LWPaHIV and may be multifactorial in origin [97]. In children, CLD has been reported in relation to lymphocytic infiltrative pneumonitis, chronic infection and other non-infectious causes, such as immune reconstitution inflammatory syndrome [98]. Features include chronic cough, shortness of breath, reduced exercise tolerance and frequent respiratory tract infections [99]. In a study of 116 adolescents LWPaHIV aged 10–19 years in Zimbabwe, 40% had hypoxia either at rest or during exercise, with abnormal pulmonary function tests and chest x-ray (CXR) findings in 45% and 47%, respectively [99]. Common radiographic findings from 193 young people (aged 6–16 years) LWPaHIV in South Africa included ring and tramline opacities on CXR (29%) and hypoattenuation on high-resolution CT (HRCT) (36/84, 43%) [100]. In contrast, a UK study in 98 individuals LWPaHIV, with a median age of 17.9 years (IQR—14.1–21.4), found abnormalities in 47% of the CXR performed, which included ring or tramline opacities in 19% [101]. A diagnosis of bronchiectasis or bronchiolitis obliterans was made in 8% of individuals. Overall, clinical and radiological diagnoses of CLD were higher in those born in sub-Saharan Africa, when compared to those born in the UK, which may reflect early environmental exposures and diversity in respiratory pathogens such as tuberculosis (TB) and later age at an HIV diagnosis [101]. Higher rates of fixed airway obstruction have also been reported in a US study of young people LWPaHIV (10–21 years), when compared to age-matched HEU youth, despite a similar overall prevalence of obstructive lung disease [102]. The reduced reversibility of obstructive lung disease in this population of youth LWPaHIV may indicate early chronic obstructive lung disease [102]. 

While the long-term health impacts of tobacco/cannabis smoking are well established, electronic cigarettes may also pose a significant public health concern, particularly in young people [103]. Data from the 2019 National Youth Tobacco Survey in America demonstrated that up to 27.5% of high school students (4.1 million people) self-reported e-cigarette use [104], with an increasing number of never combustible cigarette smokers. Severe respiratory effects of vaping have been reported, including e-cigarette or vaping product-use associated with induced lung injury (EVALI), which may present with acute hypoxia. Long-term health effects of e-cigarette use remain limited [103]. A summary of data gaps and recommendations for improving respiratory health is highlighted in Table 5.

## 7. Bone Health

Over half of adult peak bone mass (PBM) accrual occurs during puberty and adolescence, a period of rapid growth [106]. Low PBM is an important determinant for the development of osteoporosis in later life [106]. Prevalence estimates of low bone mineral density (BMD) in children and young people LWPaHIV range from 4% in HIC [107] to 32% in low-middle income countries [108,109,110]; these are higher rates than in youth without HIV [111]. In a UK study of 130 adults LWPaHIV, abnormal BMD (osteopenia and/or osteoporosis) was demonstrated in 44% [112]. Adverse bone health is of particular concern in adults LWPaHIV, due to the presence of multiple risk factors for low BMD in this population, including traditional risk factors (e.g., early years malnutrition, low BMI, vitamin D deficiency), HIV-related factors (e.g., advanced HIV disease, uncontrolled viral replication, chronic immune activation and inflammation) and the cumulative effects of certain ART agents known to impact bone mineralisation, such as tenofovir disoproxil fumarate (TDF) [113,114]. Accordingly, the British HIV Association (BHIVA) recommends tenofovir alafenamide as an alternative to TDF in those < 25 years of age, prior to the attainment of PBM, due to its improved bone and renal safety profile [115]. A recent systematic review of the effects of vitamin D supplementation in children and young adults living with HIV (3–25 years) suggested that supplementation increased serum 25-hydroxy vitamin D levels and, at high doses (1600–4000 IU daily), improved the total BMD [116]. However, the evidence for the benefit of vitamin D supplementation in improving BMD in people living with HIV is limited, particularly in young people LWPaHIV [117]. Alendronic acid has demonstrated both safety and efficacy for over 96 weeks in children and adolescents LWPaHIV with low BMD [118]. A summary of data gaps and recommendations for improving bone health is highlighted in Table 6.

## 8. Sexual and Reproductive Health Needs

Young people LWPaHIV face unique challenges associated with their sexual behaviour, including sharing their HIV status with new partners, as, in doing so, they potentially disclose the HIV status of their parents and siblings. Many of this group of young people have grown up with HIV stigma, and face challenges in negotiating their sexuality and their relationships while living with HIV, as well as considering the potential for onward transmission [58]. Understanding sexual and reproductive health (SRH) needs in this population is crucial for providing high-quality, youth-friendly SRH education to promote healthy relationships and prevent both sexually transmitted infections (STIs) and unintended pregnancies. Data gaps and recommendations are summarised in Table 7. 

### 8.1. Sexual Health and Contraception 

Rates of any contraception use have been reported in up to 65% of young women LWPaHIV [119], with condom use reported in 20–30% [119,120,121]. In a recent UK study of women with LWPaHIV, 34% reported a previous pregnancy; 40% resulted in termination and 19% in miscarriage [119], with rates of termination being higher in this cohort of people LWPaHIV when compared to a US cohort of all women with HIV [122]. Alongside appropriate sexual health and contraceptive counselling, long-acting reversible contraception may be a pragmatic approach to the prevention of unintended pregnancy in this cohort with complex SRH needs. To our knowledge to date, no data exist on the SRH health of transgender individuals LWPaHIV; further research is required to provide representative data in this population. 

### 8.2. Cervical Smears and Vaccination

Human papillomavirus (HPV) is one of the most common STIs globally, and persistent infection with high-risk HPV (hrHPV) subtypes can lead to anal, cervical, oropharyngeal, penile, vaginal and vulval cancers. HIV is associated with higher rates of persistent hrHPV infection and HPV-related complications, compared to people without HIV [123]. Young people LWPaHIV may be at a higher risk of persistent hrHPV infection and HPV-related complications than those who acquire HIV later in life, due to the lifelong HIV infection and immune dysregulation. A study of HPV infection in Thai and Vietnamese adolescents aged 12–24 demonstrated higher rates of persistent hrHPV infection (65% vs. 42% at week 48; 27% vs. 10% at week 144; *p* = 0.01) and abnormal cervical cytology results (33% vs. 16%; *p* = 0.01) in those LWPaHIV vs. age-matched HIV-negative controls, respectively [124]. Over a 3-year follow up period, 3 individuals developed cervical intraepithelial neoplasia (CIN) stages 2–3, all with PaHIV [124]. Whilst not disaggregated by the route of infection, cervical carcinoma has been reported in 15–19 year olds living in South Africa [22].

Highly effective strategies to prevent cervical cancer include both hrHPV vaccination and cervical screening [125]. However, the implementation of these preventive measures varies globally; despite access, low cervical screening uptake has been demonstrated in young women LWPaHIV [119]. This may be due to embarrassment and fear of physician-led screening, as well as cultural barriers [119]. In a recent UK observational study of the HPV prevalence and serostatus in women LWPaHIV with a median age of 18 years (18–34), 14/46 (30%) individuals had hrHPV subtypes detected in cervical sampling, of which 2/14 (14%) were positive for HPV16 and 0/14 for HPV18 [126]. The two individuals positive for HPV16 were either unvaccinated or had an unconfirmed vaccine status. A total of 9/12 (75%) of the remaining individuals with non-16/18 hrHPV subtypes reported a previous HPV vaccination with dual/quadrivalent vaccines, supporting the use of the nine-valent vaccine [126]. 

HPV data are limited in male adolescents LWPaHIV, although it is known that there is a high prevalence of HPV in men who have sex with men (MSM) and in MSM living with HIV [127]. A study of Thai young men LWPaHIV aged 12–24 years, with 88% reporting female-only sex, demonstrated a higher incidence and prevalence of hrHPV infection when compared to their HIV-negative peers [128]; supporting the use of hrHPV vaccination in men LWPaHIV. Further data are required to determine rates of HPV-related complications in this population.

## 9. Pregnancy in PaHIV

An increasing number of adults born with PaHIV are having children of their own, the vast majority of the third generation being born HIV-free [129]. Whilst data are emerging for women through pregnancy surveillance systems, much less is known about the pregnancy outcomes for men LWPaHIV. Early data from the US suggest similar rates of pregnancy, with 41% of women and 38% of men LWPaHIV reporting lifetime pregnancy with a partner [45]. However, data gaps for men include parenting desires, fertility, incidence, and outcomes for partners and offspring [45,130]. Several studies in Europe and the US have suggested lower rates of pregnancy in people LWPaHIV than their HIV-negative peers, although rates of unplanned pregnancy and termination remain high in some cohorts [119,129,131,132]. Despite universal ART, women living with HIV remain at an increased risk of adverse maternal and perinatal outcomes when compared to pregnant people without HIV, including preterm delivery, low birth weight and small for gestation-age infants [133,134]. Similarly, adolescent pregnancy, defined as women aged 10-19 years, is associated with increased rates of maternal morbidity, pre-eclampsia and infection, and adverse neonatal outcomes, including preterm delivery and low birth weight [135]. 

Pregnant people LWPaHIV are younger and have been exposed to more ART regimens; they are more likely to have a detectable viral load, immunosuppression and HIV-associated drug resistance mutations than those with nPaHIV acquired later in life, although obstetric outcomes are broadly similar when adjusted for age and ART regimen [28,129,136,137,138,139]. The majority of people LWPaHIV who are viraemic at conception achieve viral control by delivery; however, rates of the post-partum viral rebound are high, occurring in more than 50% in a US cohort and associated with younger age, pre-conception viraemia and immunosuppression [140]. Early data were limited to resourced settings; however, increasing data are emerging from low-middle income settings, including sub-Saharan Africa. Surveillance data from the Western Cape (*n* = 258 pregnancies) suggest a picture comparable to that observed in HIC, with high rates of terminations (14%), immunosuppression (20% with a periconception CD4 count < 200 cells/uL) and maternal viraemia (28% HIV VL > 1000 c/mL) [136]. Neonatal outcomes identified 20% as preterm (<37 weeks gestation) with 20% being of low birth weight; however, comparable national data were not available and 39% of pregnancies occurred in very young people LWPaHIV, aged 16 years or younger. A total of 2.2% of infants were known to be infected, which is reassuringly comparable to the national vertical transmission rate in South Africa of 2.4%, although, more worryingly, 22% of infants were untested. Data from Botswana comparing pregnancy outcomes in PaHIV (402) and nPaHIV (8465) matched for the maternal age range (15–27 years, with a median age of PaHIV 20 years; nPaHIV of 24 years) suggested higher rates of small for gestational age infants born to those LWPaHIV, although, when adjusted for an ART regimen, outcomes were similar [137]. 

Whilst rates of transmission are reassuringly low, longer-term outcomes for HIV-exposed uninfected third-generation infants born to people LWPaHIV are yet to emerge. HEUs born to people LWnPaHIV have increased mortality and morbidity, with impacts on growth and early infant development when compared to their HIV-unexposed peers, due multiple factors that are not limited to poor maternal health, ART exposure and socioeconomic risk factors [141]. People LWPaHIV are younger mothers with poorer virological and immunological parameters compared to their nPaHIV counterparts, with many having lost their own parents and living in economic hardship with limited social support [45]. Early data suggesting modest differences in the early development and increased risk of infectious morbidity for HEUs born to mothers LWPaHIV compared to nPaHIV and highlights the need for follow up of this specific cohort, as they join the global population of more than 15 million HEUs born to women who have acquired HIV later in life [142,143,144].

## 10. Discussion

**Figure 1:**
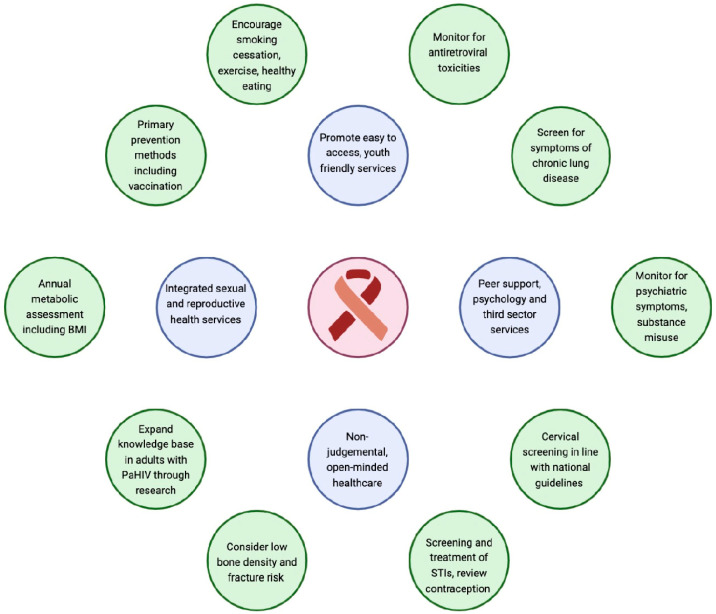
Providing optimal care and health outcomes for adults perinatally infected with HIV. (Created with Biorender.com).

In this review article, we summarise the current knowledge of clinical outcomes for a unique cohort of young people growing up with PaHIV, the majority of whom live in less developed settings with a high burden of HIV disease. To provide optimal care and health outcomes for these individuals, addressing the specific health needs of this population is crucial (Figure 1).

The complex issues that this group of young people faced as they grew up with HIV are different from those who acquired HIV as adults. Whilst many healthcare settings have access to “adolescent corners”, the specific emotional needs of the perinatal group may differ significantly to other young people who acquired HIV behaviourally. There is a disproportionate link with migration, poverty, orphanhood, becoming a child carer for unwell family members, HIV-associated stigma and having to negotiate the first and every subsequent sexual encounter in the context of a stigmatised and sexually transmissible condition. Where resources are available, appropriate psychological supportive interventions need to be tested and crafted, with young people driving the agenda to enable them to feel empowered, engaged and able to navigate their lifelong health and well-being. Working closely with the community of young adults growing up with HIV, the current research is looking at developing tools that can best deliver such support. The link with HIV-stigma, lack of parental support and poor health education has been shown to link directly with poor linkage into care, sub-optimal ART adherence, consequent viral failure and risk of transmission, at a time when most people are their most sexually and reproductively active. 

To more carefully evaluate the current data gaps in clinical outcomes and risk of complications, it is reasonable to divide the current cohorts of adults with perinatally acquired HIV into two main groups; firstly, (cohort 1) those who survived the pre-ART period as children, born before 2000, after which paediatric ART formulations became more widely available, and the second group (cohort 2), who are now >18 years old and many of whom have been treated with antiretroviral therapy for the majority of their lives. The first group are enriched with genetic and immunologically protective factors, and those living in better resourced settings may have had access to services able to support serious health complications, prior to accessible paediatric ART. While the second group may also reflect these characteristics, the majority will have had a lifelong exposure to ART with shifts in ART availability and guideline recommendations. 

There are several potential mechanisms that may be involved in driving long-term health complications amongst young people growing up with HIV. These include, (1) immunological: the consequence of a developing immunological maturity in the presence of a CD4 tropic virus, conferring immune dysfunction, failure of immune maturation, thymic infection and dysfunction, immune exhaustion and the development of auto-immunity [145]; (2) virological: direct virally mediated inflammation end organ damage, which is more significant for those with an uncontrolled viral replication during organ maturation, in particular for vulnerable sites such as the brain, heart, lungs and kidneys [16]; (3) antiretroviral toxicities: the dosing of medication during childhood is often suboptimal, due to changing weights leading to periods of sub-therapeutic or toxic dosing with sequelae that become apparent in later years [146,147]. This might be relevant to early protease inhibitor-based ART regimens used in children with a consequent impact on weight gain, lipodystrophy, metabolic syndrome and bone density changes. The extrapolation of new findings from intervention studies in adults, such as the recently published REPRIEVE study [148], which demonstrated a significant reduction in cardiovascular disease following the use of pitavastatin, as compared with standard care, will need a re-assessment in the context of an adult perinatal cohort. 

The data highlighted in this review are synthesized from multiple sources, countries, cultures and health systems, and care needs to be taken in extrapolating data from one context to another, with consideration of local factors being important, particularly with changing access to ART, mental health and quality of life. However, signals from many varied settings highlight the emerging concerns for cardiovascular, metabolic and respiratory health for adults living with PaHIV, the consequences of which may only emerge clinically in mid-adult life. To address the multitude of unanswered questions for this unique population requires a robust global data linkage as adolescents transition into adult care. Further multimodal intervention studies are required to improve the retention in care and adherence to ART, including the use of emerging long-acting therapies in vulnerable non-suppressed youth. However, this pathway starts in infancy, as the consequences of very early life experiences, including access to early infant HIV diagnosis and ART, and their impact on physical health and cognitive, emotional and social functioning in adult life, cannot be underestimated.

## 11. Conclusions

Comprehensive, collaborative research on the clinical characterisation of adult perinatal cohorts, globally, has the capacity to define key clinical complications and explore the underlying mechanisms that may be involved. A careful robust collation of these data will inform the best practice to optimise long-term outcomes.

## Figures and Tables

**Table 1:** Summary and Recommendations for Cascade of Care.

Monitor	Management	Data Gaps
Engagement in care	Youth-friendly services removing barriers to accessing care within local service provision	Accurate linkage of paediatric and adult data sets where data are disaggregated by route of transmission (PaHIV versus nPaHIV) How best to re-engage those who have disengaged from care? Effective adherence interventions for young adults, including cost effectiveness?
Mortality	Maintaining engagement in care and access to integrated services during transition to adult care. Optimising viral suppression	

**Table 2:** Summary and Recommendations for Viral Suppresion and Acquired Drug Resistance.

Monitor	Management	Data Gaps
Viral load	Resistance sequence in virological failure	Emergent incidence of integrase resistance with wide-spread global use of dolutegravir Optimal ART sequencing where integrase resistance occurs Potential for use of long-acting ART in non suppressed youth
Adherence	ART optimisation to single tablet regimens where possible	

**Table 3:** Summary and Recommendations for Neurocognitive, Mental Health and Quality of Life.

Monitor	Management	Data Gaps
Mental health at each clinic visit	Access to integrated mental health services and peer support	Rates of psychosis in adults LWPaHIV; how does this compare with the general population, HEU young adults and those LWnPaHIV? What are the causes of adverse mental health outcomes and the role of HIV-related factors, such as inflammation versus traditional risk factors? Neurocognitive outcomes in adult life; impact of quality of life and brain aging in 5th decade and beyond
Substance use at each clinic visit	Advice and information around substance misuse starting in early adolescence Early referral to cessation services when substance use is identified	

**Table 4:** Summary and Recommendations for Metabolic and Cardiovascular Health.

Monitor	Management	Data Gaps
Weight/BMI at each clinic visit	If they are overweight, provide lifestyle advice and refer to the dietician Review ART regimen	Long-term risks associated with obesity and chronic inflammation; how does this compare with the general population? What is the role of ART versus traditional risk factors towards CVD? Is there an increased risk of CVD in this group and what is the mechanism? What are the optimal CVD interventions to avoid complications and polypharmacy?
Blood pressure at each clinic visit	If there is raised blood pressure, refer to hypertension guidelines for further investigation and management	
Metabolic assessment: Annual Hba1c and lipids	If abnormal, provide lifestyle advice, refer to the dietician and where appropriate, provide treatment	
Monitor liver function every 6 months	If abnormal, non-invasive liver screening including ultrasound and fibroscan for NAFLD	
Selected high-risk groups for enhanced monitoring; e.g., hypertensive, high BMI, high lipids and smokers	Where available, refer to a CVD clinic for enhanced monitoring and intervention	
Monitor for alcohol misuse	Refer to support services	

**Table 5:** Summary and Recommendations for Respiratory Health.

Monitor	Management	Data Gaps
Screen for symptoms of CLD—shortness of breath, cough and/or sputum and wheezing [105]	Refer for spirometry. If abnormal, provide referral to respiratory specialist, +/− CT imaging. Assess for concomitant chronic diseases.	Unknown long-term progression of CLD Research towards novel biomarkers to understand mechanisms of CLD
Exacerbations of CLD	Sputum cultures (MC + S; TB). Consider prophylactic antibiotics and rescue packs, as per national guidelines for the management of CLD. Annual influenza vaccine. COVID-19/pneumococcal vaccine, as per national guidance	
Smoking and/or vaping use	Smoking cessation services	Data to determine the risk of health impacts in people with PaHIV compared to the age-matched HIV-negative population

**Table 6:** Summary and Recommendations for Bone Health.

Monitor	Management	Data Gaps
Fracture risk	Entry to adult care—consider baseline dual-energy X-ray absorptiometry scan in those with additional risk factors such as reduced mobility. If abnormal, refer to osteopenia/osteoporosis guidelines and bone specialist.	Future fracture risk in adults with PaHIV and its association with low BMD, vitamin D and ART
Annual blood vitamin D and calcium levels	Replacement if deficient	

**Table 7:** Summary and Recommendations for Sexual and Reproductive Health.

Monitor	Management	Data Gaps
HIV RNA	SRH education Peer support PrEP for partners if there is a viral rebound	SRH in transgender people with PaHIV STI rates in people with PaHIV Duration of immunity following HPV vaccination and need for booster doses Role of early HPV screening for people with a cervix with PaHIV HPV persistence and complications in young men with PaHIV Developmental outcomes for HEUs born to people LWPaHIV
STI/blood-borne virus rates	SRH education Condom use Treatment of STIs Vaccination—HPV/Hepatitis A+B, M Pox	
HPV-associated cervical dyplasia and cancers	National cervical screening Referral to colposcopy as per national/local guidelines	
Pregnancy surveillance	Referral for specialist HIV antenatal care where available Unintended pregnancies—referral for abortion and contraceptive services	
Pregnancy and birth outcomes	Peer support and HIV antenatal care HIV RNA suppression	

## Data Availability

Not applicable.
